# The Role of Autophagy in Lamellar Body Formation and Surfactant Production in Type 2 Alveolar Epithelial Cells

**DOI:** 10.7150/ijbs.64285

**Published:** 2022-01-01

**Authors:** Xiaoman Li, Liang Wang, Jialin Hao, Qingfeng Zhu, Min Guo, Changjing Wu, Sihui Li, Qiqiang Guo, Qiuhong Ren, Ning Bai, Fei Yi, Bo Jiang, Wenyu Zhang, Yanling Feng, Hongde Xu, Han Jiang, Xiaoyue Zhai, Guohua Zhang, Hong-long Ji, Xuesong Yang, Dan Zhang, Jianhua Fu, Jianjun Chang, Xiaoyu Song, Liu Cao

**Affiliations:** 1 College of Basic Medical Sciences, China Medical University, Shenyang, China.; 2 Key Laboratory of Medical Cell Biology, Ministry of Education, China Medical University, Shenyang, China.; 3 Institute of Health Sciences, China Medical University, Shenyang, China.; 4 Department of Pathology, College of Basic Medical Sciences, China Medical University, Shenyang, China.; 5 Department of Vascular Surgery, The First affiliated Hospital, China Medical University, Shenyang, China.; 6 Department of Forensic Pathology, School of Forensic Medicine, China Medical University, Shenyang, China.; 7 Department of Cellular and Molecular Biology, University of Texas Health Science Center at Tyler, Tyler, USA.; 8 Institute of Biomedicine, National Engineering Research Center of Genetic Medicine, Jinan University, Guangzhou, China.; 9 Department of Pediatrics, Shengjing Hospital of China Medical University, Shenyang, China.

**Keywords:** autophagy, *Atg7*, lamellar body, type 2 alveolar cells, LC3B, surfactant protein B

## Abstract

The lamellar body (LB), a concentric structure loaded with surfactant proteins and phospholipids, is an organelle specific to type 2 alveolar epithelial cells (AT2). However, the origin of LBs has not been fully elucidated. We have previously reported that autophagy regulates Weibel-Palade bodies (WPBs) formation, and here we demonstrated that autophagy is involved in LB maturation, another lysosome-related organelle. We found that during development, LBs were transformed from autophagic vacuoles containing cytoplasmic contents such as glycogen. Fusion between LBs and autophagosomes was observed in wild-type neonate mice. Moreover, the markers of autophagic activity, microtubule-associated protein 1 light chain 3B (LC3B), largely co-localized on the limiting membrane of the LB. Both *autophagy-related gene 7* (*Atg7*) global knockout and conditional* Atg7* knockdown in AT2 cells in mice led to defects in LB maturation and surfactant protein B production. Additionally, changes in autophagic activity altered LB formation and surfactant protein B production. Taken together, these results suggest that autophagy plays a critical role in the regulation of LB formation during development and the maintenance of LB homeostasis during adulthood.

## Introduction

Type 2 alveolar epithelial cells (AT2) in the lung tissue play a critical role in retaining the alveoli's integrity and function. AT2 cells contain a unique organelle called the lamellar body (LB), a concentric structure loaded with surfactant proteins and phospholipids 1. LBs are secreted into the air space by exocytosis, where they unravel into a tubular network (tubular myelin) that is absorbed and spread at the air-liquid interface to form a phospholipid film 2. The origin of LBs has not been fully elucidated. The well-accepted view that LBs are derived from a multivesicular body (MVB) is supported by evidence from transmission electron microscopy (TEM) studies showing the transition from MVB to LB 3-6. Autoradiography studies of murine AT2 cells from mouse lungs have shown that although proteins that were metabolically labeled with ^3^H leucine were visualized in MVBs before delivery to LBs, phospholipids labeled with ^3^H choline were delivered directly to LBs 7, 8. Therefore, although surfactant proteins may be supplied by MVBs, phosphatidylcholine is not. Notably, glycogen is an established substrate for surfactant phospholipid production during lung development because glycogen depletion correlated with phospholipid synthesis 9-14.

Surfactant proteins are closely related to the formation of LBs, and when secreted into the alveolar cavity with surfactant phospholipids, they play a pivotal role in the formation and maintenance of the surfactant film 15-17. Out of four surfactant proteins (SFTPA, SFTPB, SFTPC, and SFTPD), SFTPB and SFTPC are hydrophobic and contribute to the surface tension-lowering activity 18. Only SFTPB is essential for LB formation, postnatal viability, and breathing 19-22. SFTPB-deficient mice are not viable, and consistently, infants carrying homozygous SFTPB mutations die of respiratory failure after birth 21, 22. A previous study has shown that mice may exhibit perinatal lethality when the SFTPB level is reduced to 25% of the normal level 23.

Weibel-Palade bodies (WPBs) of endothelial cells, the melanosomes of melanocytes, the LBs of AT2 cells, and the dense bodies of platelets are all lysosome-related organelles (LROs). Autophagy regulates the formation of several LROs, including WPBs and melanosomes 24, 25. Hermansky-Pudlak Syndrome (HPS), an autosomal recessive disease, is characterized by clinical manifestations of hypopigmentation, abnormal platelets, and pulmonary fibrosis, which are all coincidentally related to LROs 26. Cells from lung fibrosis patients suffering from HPS show altered autophagy and abnormal LBs 27, 28. Taken together, it is plausible to speculate that autophagy is involved in regulating the homeostasis of another LRO, the LB. Although LB formation was compromised by treatment with the autophagic inhibitor 3-methyladenine (3-MA) in a cellular model 8, 15, 29, the detailed mechanism of how autophagy regulates the formation of LBs has not been fully elucidated in an *in vivo* autophagy-deficient model.

In the present study, we demonstrated that autophagy is an essential physiological process for regulating LB formation and maintaining the proper function of AT2 cells. Mice with global deletion of *autophagy-related gene 7* (*Atg7*) show abnormal lung morphology, impaired autophagic activity, smaller/fewer LBs, and reduced surfactant protein B. Additionally, deletion of *Atg7* in AT2 cells using the *SPC*-specific promotor produced similar effects to those observed with the global knockout of* Atg7* at the neonatal stage but led to large and irregular vacuoles (LB-like vacuoles) and degenerative AT2 cells during adulthood. In summary, our results indicate that autophagy plays a critical role in regulating the formation of LBs during development and maintaining LB homeostasis during adulthood in AT2 cells.

## Results

### Changes in autophagic activity alter LB formation and surfactant protein production

**Figure 1:**
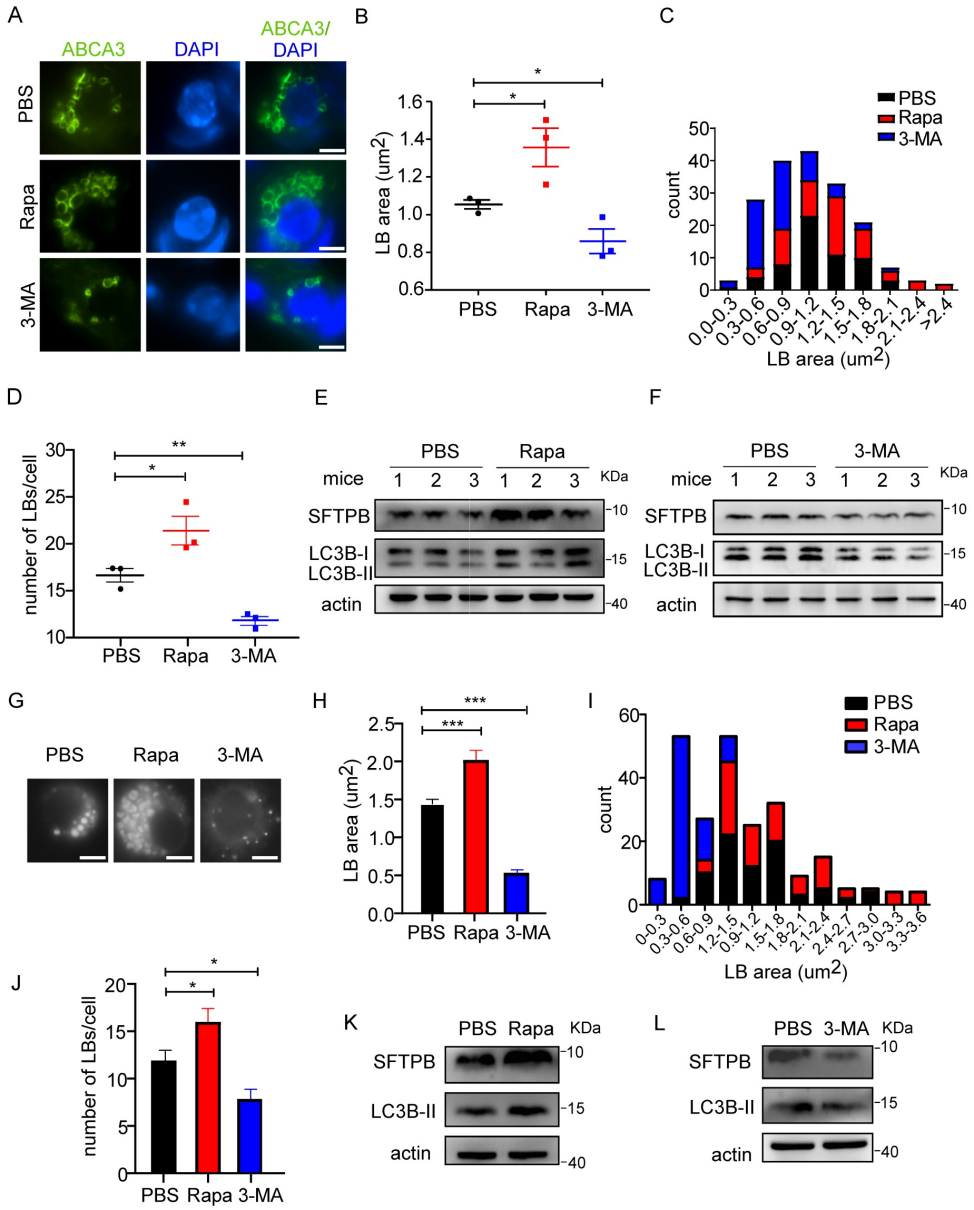
** Changes in autophagic activity alter LB formation and surfactant protein production. (A)** Immunofluorescent staining of frozen sections from control (PBS), rapamycin (Rapa), and 3-MA-treated mice. The limiting membrane of LBs was detected using an anti-ABCA3 antibody (scale bar: 5 µm). Representative AT2 cells are shown. The average area **(B)** and the size distribution **(C)** of LBs in PBS, Rapa, or 3-MA-treated mice are shown (the area of total 80 LBs in at least 5 random magnification fields per mouse was measured using ImageJ software; n = 3 mice/group). **(D)** The average number of LBs in Rapa, 3-MA, or PBS-treated mice (at least 25 random AT2 cells were chosen per mouse; n = 3 mice/group). The expression level of SFTPB in mice treated with Rapa **(E)** or 3-MA **(F)** (n = 3 mice/group; experiments were duplicated three times; representative results are shown). **(G)** Primary human AT2 cells treated with control, Rapa, or 3-MA were incubated with LysoTracker to stain LBs (scale bar: 5 µm). The experiments were repeated three times; representative AT2 cells are shown. The average area **(H)** and size distribution **(I)** of LBs from Rapa or 3-MA-treated primary human AT2 cells were plotted compared with controls (80 random LBs were measured/group). **(J)** The average number of LBs in Rapa, 3-MA, or PBS-treated primary human AT2 cells (n ≥25 AT2 cells/group). The expression level of SFTPB in primary human AT2 cells treated with Rapa **(K)** or 3-MA **(L)** (experiments were duplicated three times; representative results are shown). *P < 0.05; **P < 0.01; ***P < 0.001.

To test whether altered autophagic activity affects LB formation, an autophagy inducer and an inhibitor were used. Rapamycin, which enhances autophagic activity by inhibiting the mechanistic target of rapamycin kinase (mTOR)-UNC-51-like kinase 1 (ULK1) pathway 30, 31, was administrated to 8-week-old WT mice, and lungs were harvested and analyzed 8 hours later. Upon rapamycin treatment, the size of LBs was increased (Figure 1A, B), as demonstrated by the immunofluorescent staining of ATP-binding cassette sub-family A (ABC1), member 3 (ABCA3), which is localized on the limiting membrane of the LB 32. The size distribution of LBs in control and rapamycin-treated mice is shown in Figure 1C. The number of LBs was also significantly increased upon treatment with rapamycin (Figure 1D). Consistent with the downregulation of mTOR signaling by rapamycin, we observed an increased level of LC3B-II (Figure 1E). Furthermore, the expression level of SFTPB was elevated upon rapamycin treatment (Figure 1E). To further elucidate the role of autophagy in LB formation, we administered 3-MA, which blocks the phosphatidylinositol 3-kinase (PI3K) pathway and inhibits autophagy, or vehicle control to WT mice. 3-MA treatment decreased both the size and number of LBs (Figure 1A-D). Consistently, western blot analysis showed that the SFTPB level was downregulated upon 3-MA treatment, and the extent of autophagy inhibition was determined by measuring the LC3B-II level (Figure 1F).

We further studied the effects of autophagy on LBs in isolated primary human AT2 cells. LysoTracker, a fluorescent probe for tracking acidic organelles in live cells, was used to label LBs in AT2 cells. Consistent with the results observed in AT2 cells from mice, rapamycin increased the number and size of LBs (Figure 1G-J) and the level of SFTPB in human AT2 cells (Figure 1K). Conversely, treatment with 3-MA significantly reduced both the number and size of LBs (Figure 1G-J). Moreover, the level of SFTPB was decreased by 3-MA treatment (Figure 1L). Taken together, these results suggest that autophagy regulates LB formation and surfactant protein production in both mice and human AT2 cells.

### Atg7-deficient mice show defects in lung development, LB formation, and surfactant biogenesis

**Figure 2:**
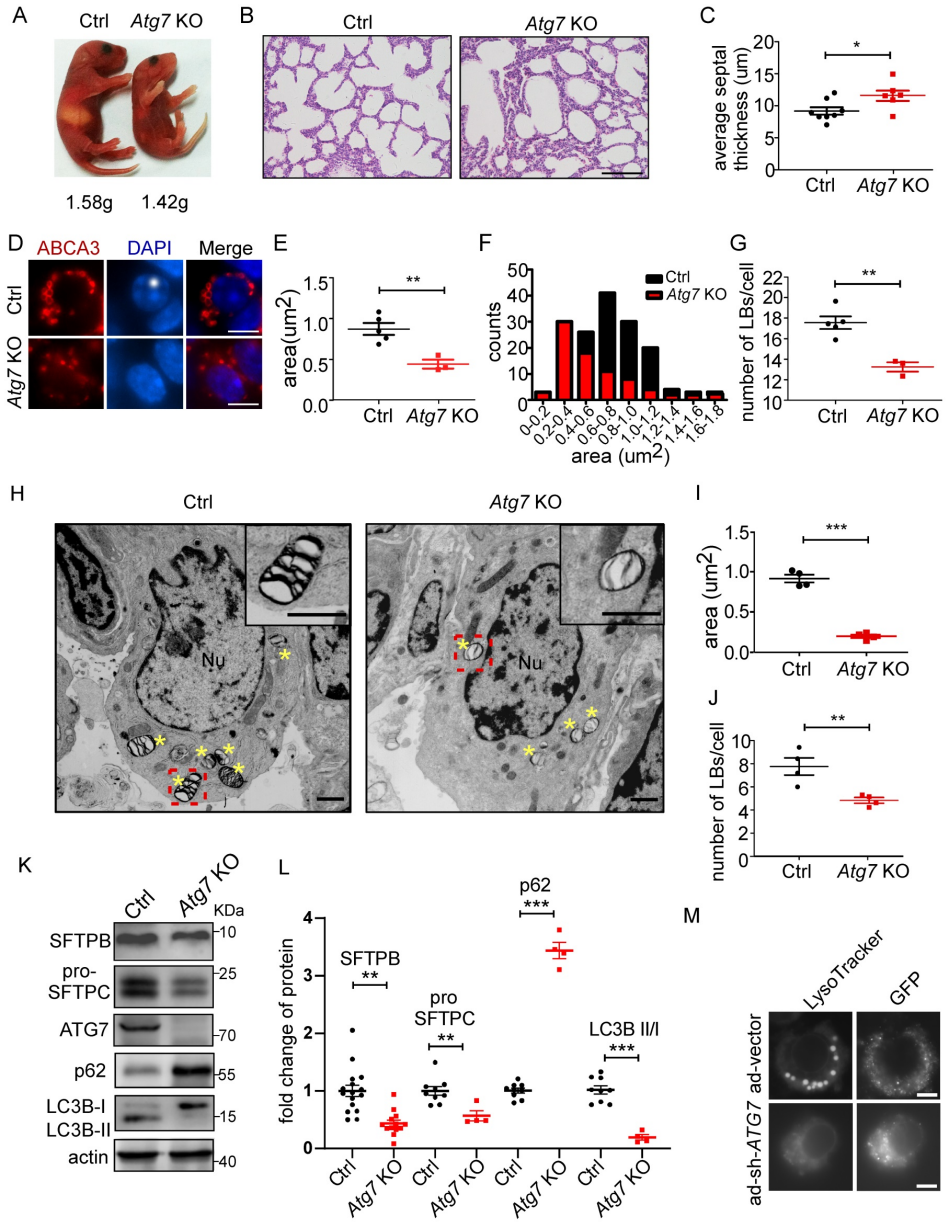
** Atg7-deficient mice show defects in lung development, LB formation, and surfactant production. (A)** Gross appearance of representative neonates. The bodyweight of Atg7^-/-^ mice was slightly but significantly lower than that of wild-type (WT) mice (1.42g vs. 1.58g; n = 5). **(B)** Histological analysis of lungs from WT control (Ctrl) and Atg7 KO neonates (scale bar: 100 µm). **(C)** Septal thickness was measured at all points in each visual field (at least five random visual fields in high magnification per mouse were used for measurements; WT, n = 8, Atg7 KO, n = 6). **(D)** Frozen sections of Ctrl and Atg7 KO neonate lungs were stained with the anti-ABCA3 antibody to detect LBs (scale bar: 5 µm). Representative single cells are shown. The average area **(E)** and size distribution **(F)** of LBs were quantified by ImageJ software (80 LBs/mouse in at least five random high magnification fields were measured; WT, n = 5; Atg7 KO, n = 3). **(G)** The average number of LBs per cell was quantified (at least 25 AT2 cells/mouse were quantified; WT, n = 5; Atg7 KO, n = 3). **(H)** Electron microscopy analysis shows a marked reduction in the quantity and quality of LBs in Atg7 KO mice compared with controls (* denotes LB; Nu denotes nucleus; Insets are enlarged LBs; scale bar: 1 µm). **(I)** Quantification of the average area of LBs (n = 4 mice/group). **(J)** The number of LBs per AT2 cell in each genotype (n = 4 mice/group). **(K)** The levels of SFTPB and pro-SFTPC in extracts of whole lung tissue from Ctrl and Atg7 KO mice were determined by western blotting. Experiments were repeated more than three times; representative images are shown. **(L)** Densitometry analysis of SFTPB, pro-SFTPC, p62, and LC3B II/I in Ctrl and Atg7 KO mice (for SFTPB, WT, n = 16, Atg7 KO, n = 13; for pro-SFTPC, p62, and LC3B II/I, WT, n = 9, Atg7 KO, n = 4). **(M)** Knockdown of *ATG7* by adenoviral infection inhibited LB formation. Cells infected with ad-vector have both lysotracker staining and GFP-fluorescence; cells infected with ad-sh-*ATG7* have GFP-fluorescence but lack lysotracker staining (scale bar: 5 μm). *P < 0.05; **P < 0.01; ***P < 0.001.

ATG7, a component of the ATG conjugation systems, is pivotal to the expansion and closure of autophagosome membranes. Targeted deletion of *Atg7* leads to perinatal lethality, which may be due to the non-suckling phenotype in the early neonatal period 33, 34. *Atg7*-deficient mice (*Atg7* KO) died within 12 h, whereas non-suckling littermate controls died approximately 24 h after birth. Therefore, we analyzed the morphological development of lungs and LB formation in *Atg7^-/-^* mice (P1). The gross appearance of *Atg7* KO mice indicated their size was smaller than that of wild-type (WT) mice (Figure 2A). The bodyweight of *Atg7* KO mice (1.42 g) was slightly lower than that of WT mice (1.58 g). Lungs from *Atg7* KO mice and WT littermates were harvested as soon as the pups were found in the breeding cage. Hematoxylin and eosin (H&E) staining showed that *Atg7* KO mice had unevenly expanded air sacs and impaired thinning of the alveolar septa. In contrast, the lungs of WT neonates had normal air sac expansion and septal thinning at birth (Figure 2B, C).

Next, we examined whether the *Atg7* deletion in mice compromised the formation of LBs. We found that the LBs in *Atg7* KO mice were significantly smaller than those in WT mice (Figure 2D-F and Supplemental Figure 1A-C). Frequently, the hollow circle delineated by ABCA3 staining in WT mice was absent in *Atg7* KO mice. Moreover, the number of LBs in* Atg7* KO mice was lower than in WT mice (Figure 2G). Similarly, electron microscopy (EM) revealed that the abnormal LBs in *Atg7* KO mice contained fewer lamellae than those in WT mice (Figure 2H). EM images also confirmed a reduction in LB size in *Atg7* KO mice, compared with the WT controls (Figure 2I). The analysis of random sections showed that the mean number of LBs per AT2 cell was also dramatically reduced (Figure 2J), further supporting the notion that ATG7 may be involved in the growth of LBs.

The surfactant proteins SFTPB and pro-SFTPC were reduced in *Atg7* KO mice (Figure 2K and L and Supplemental Figure 2A, B), further supporting the idea that LBs were compromised in *Atg7* KO mice. However, SFTPA was unaltered (Supplemental Figure 2C), consistent with previous reports that SFTPA was secreted in an LB-independent manner 35-37. The *SFTPB* and *SFTPC* promoters are reportedly regulated by thyroid transcription factor-1 (TTF-1/Nkx2.1) in AT2 cells 38. However, TTF-1 expression in *Atg7* KO mice was no different from that in WT mice (Supplemental Figure 2D). Next, we assessed autophagic activity by determining the ratio of the expression levels of LC3B-II and LC3B-I and the amount of the autophagy substrate, SQSTM1 (referred to here as p62). In *Atg7* KO lungs, the LC3B-II/LC3B-I ratio was reduced compared with the controls (Figure 2K, L), consistent with ATG7 being essential for ATG conjugation systems and autophagosome formation. Western blot analysis also showed the accumulation of p62 in *Atg7*-deficient lungs compared with the controls (Figure 2K, L). Because AT2 cells constitute only a small percentage of the cell population in the lung, we isolated primary AT2 cells by magnetic-activated cell isolation and subjected them to western blot analysis. Both SFTPB and pro-SFTPC levels were further reduced in the AT2 cells compared with the whole tissue lysate (Supplemental Figure 3A, B). To directly assess the effect of *ATG7* on LBs, we infected human primary AT2 cells with adenoviruses carrying sh-*ATG7*-EGFP to knockdown endogenous ATG7. Consistent with the previously presented results, ablation o of *ATG7* compromised LB formation in human primary AT2 cells (Figure 2M, Supplemental Figure 4). Lung surfactant comprises 80% glycerophospholipids, 10% cholesterol, and 10% surfactant proteins. Phosphatidylcholine (PC) and phosphatidylglycerol (PG) are the predominant phospholipids of LBs and make up 70%-80% and 5%-10%, respectively 39. The primary form of PC is its unsaturated form as dipalmitoyl-phosphatidylcholine (DPPC; 16:0/16:0). To examine whether *Atg7* deletion affects the lipid content of LBs, we subjected whole-lung homogenates to quantitative lipid chromatography-tandem mass spectrometry (LC-MS/MS). Among 949 different lipids detected, no significant difference was found in the most abundant lipids, including DPPC and cholesterol. By our criteria, only 14 phosphatidylglycerols were downregulated in the *Atg7* KO lung (Supplemental Figure 5 and Supplemental Table 1). Taken together, the present study indicates that *Atg7* is critical for lung development, LB formation, and surfactant SFTPB/C production.

### The role of autophagy in LB formation

**Figure 3:**
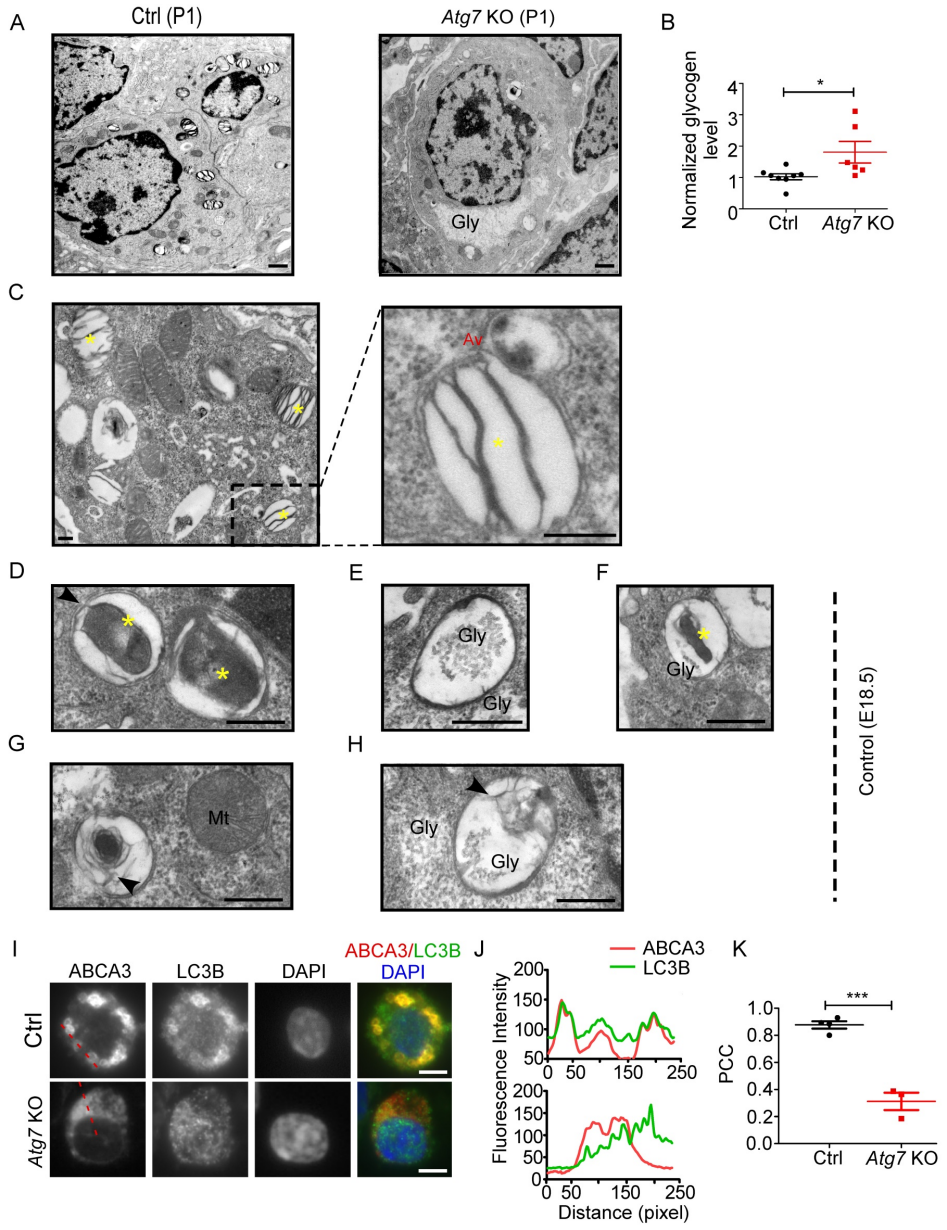
** Autophagic vacuoles are involved in LB formation. (A)** EM analysis shows the abnormal glycogen accumulation in Atg7 KO P1 neonate mice (Gly denotes glycogen, scale bar: 1 µm). **(B)** Glycogen levels in lung homogenates from Atg7 KO mice and littermate controls (mice/group WT, n = 8; KO, n = 6). **(C)** Electron micrographs of AT2 cells from WT neonatal mice (P1) showing LBs fusing with autophagosomes (* denotes LB; Av denotes autophagic vacuole; scale bar: 200 nm). Electron micrographs of AT2 cells from an E18.5 WT mouse lung with autophagosomes containing cytoplasmic contents **(D),** glycogen particles **(E),** and a combination of cytoplasmic contents and undigested glycogen particles **(F)** (* denotes cytoplasmic contents/organelles, Gly denotes glycogen; scale bar: 500 nm). The lamellar transition from autophagosome could be seen in **(G) and (H)** (Mt denotes mitochondria, arrowhead denotes the appearance of lamellae; scale bar: 500 nm). **(I)** Co-localization of ABCA3 and LC3B in WT and Atg7 KO mice (scale bar: 5 µm). **(J)** Line profile plot of the immunofluorescence (red line) is shown. **(K)** Pearson correlation coefficient (PCC) for WT and Atg7 KO mice (mice/group WT, n = 4, KO, n = 3) was calculated. *P < 0.05, ***P < 0.001.

Because the enrichment of glycogen is characteristic of immature AT2 cells 14, we examined the glycogen level in *Atg7* KO and control mice. EM revealed that more glycogen-filled AT2 cells were present in P1 *Atg7* KO mice than in littermate controls (Figure 3A). Consistently, the glycogen levels, measured by the amount of glucose hydrolyzed from glycogen 40, were higher in* Atg7* KO mice than those in littermate controls (Figure 3B). Periodic acid-Schiff (PAS) staining, which stains carbohydrate macromolecules such as glycogen and glycoproteins, was also performed on lung sections. Consistently, the PAS-staining intensity was higher in* Atg7* KO mice than in WT controls (Supplemental Figure 6A). Moreover, PAS staining co-stained with ABCA3, suggesting that glycogen mainly accumulated in AT2 cells (Supplemental Figure 6B). Collectively, the abnormally accumulated glycogen in the AT2 cells of *Atg7* KO neonate lungs suggests delayed differentiation of AT2 cells.

To understand how autophagy is related to LB formation, we analyzed electron micrographs of neonate WT mice (P1). When applying the routine EM fixation procedure, autophagosomes in AT2 cells are characterized by a double membrane enclosing regions of low electron density or an electron-translucent cleft 41. LBs and autophagosomes were often found in close proximity to each other within AT2 cells. Moreover, we observed multiple instances in which an LB actively fused with an autophagosome in P1 WT neonates (Figure 3C). Because both the glycogen content and the autophagic activity peak prior to term (around E18.5) and fall by birth 42, 43, we also examined the electron micrographs of E18.5 *Atg7* KO and WT mice. Interestingly, we readily observed autophagosomes enclosing cytoplasmic contents, including glycogen particles (Figure 3D-F and Supplemental Figure 6C), in the lungs of E18.5 WT mice. The appearance of lamella transitioning from cytoplasmic contents and glycogen was also observed (Figure 3G, 3H). Although glycogen was sequestered in E18.5 *Atg7* KO mice, the characteristic autophagic membrane was not observed (Supplemental Figure 6D). Taken together, these data suggest that autophagy may be involved in LB biogenesis during development by engulfing and using cytoplasmic contents such as glycogen. Moreover, autophagy may maintain the LB function by continuous fusion between autophagosomes and LBs.

To further confirm the autophagic origin of LBs, we performed double immunofluorescence using anti-ABCA3 and anti-LC3B antibodies. Interestingly, we found that LC3B and ABCA3 largely colocalized on the LB membrane in WT neonate mice (Figure 3I, J, upper panel). The colocalization of LC3B and ABCA3 was as high as 0.88, as measured by Pearson's correlation coefficient (PCC) (Figure 3K). However, in *Atg7* KO mice, LC3B was dispersed (Figure 3I, J, lower panel), and the PCC was 0.31 (Figure 3K). Collectively, these observations suggest that autophagy is essential for LB formation and that the LB membrane, at least in part, is derived from the autophagosome.

### Lung development defects and altered LB formation in bronchioalveolar epithelium-specific Atg7 deletion mice

To further elucidate the function of ATG7 in AT2 cells, bronchioalveolar epithelium-specific *Atg7* KO mice were generated. Mice harboring floxed *Atg7* alleles were mated with *SPC-rtTA* mice that express the *rtTA* gene under the control of the *SFTPC* promoter (hereafter referred to as *Atg7^SPC^* mice). *Atg7^SPC^* pups did not show neonatal lethality and exhibited no cyanosis or growth retardation. The postnatal growth of *Atg7^SPC^* mice was also comparable to that of the control mice.

**Figure 4:**
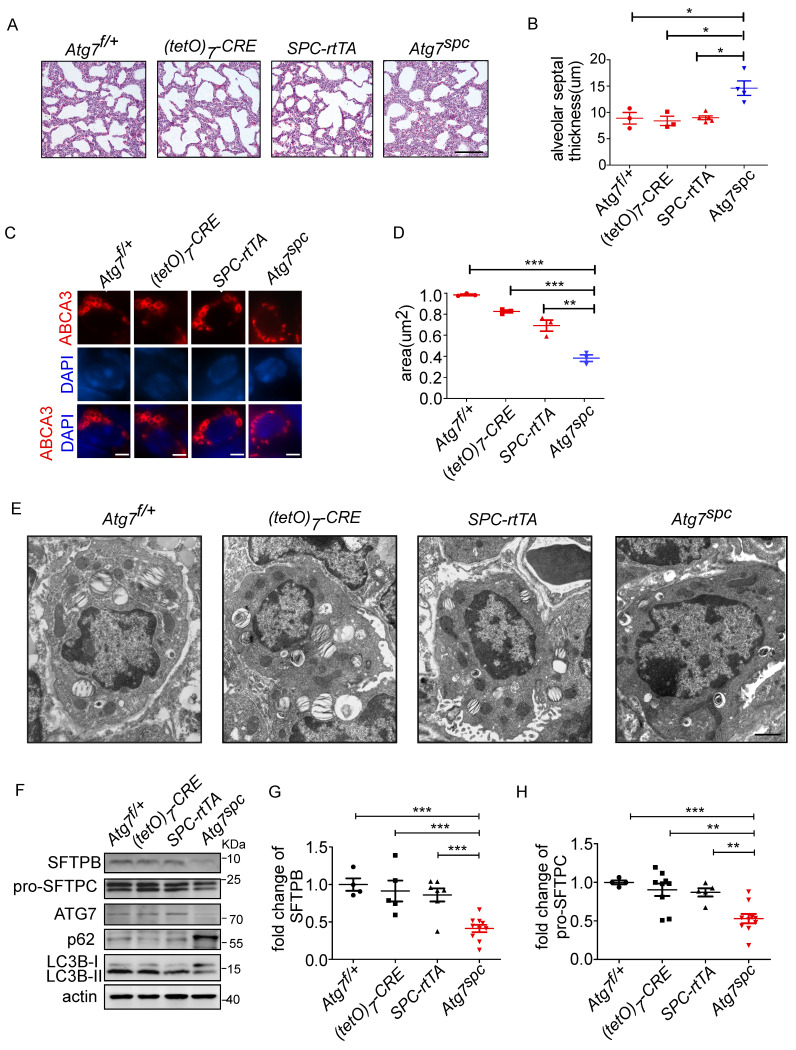
** Lung dysplasia and abnormal LBs are observed in bronchioalveolar epithelium-specific Atg7 conditional knockout mice. (A)** Histological analysis of neonatal lungs from Atg7^flox/+^, Atg7^flox/+^:(tetO)7-Cre, Atg7^flox/+^:SFTPC-rtTA, and Atg7^SPC^ neonates (scale bar: 100 µm). **(B)** Septal thickness was measured at all points in each visual field, and five visual fields for each animal were used for the measurements (n ≥3 mice/group). **(C)** Frozen sections of Atg7^flox/+^, Atg7^flox/+^:(tetO)7-Cre, Atg7^flox/+^:SFTPC-rtTA, and Atg7^SPC^ neonates' lungs were stained using anti-ABCA3 antibody to detect LBs (scale bar: 2.5 µm). **(D)** The average size of LBs in control and Atg7^SPC^ neonate lungs (≥80 LBs in at least 5 ransom high magnification fields were measured/mouse, mice/group n = 3). **(E)** EM analysis shows robust LBs in the control mice, whereas those in Atg7^SPC^ mice contain few lamellae (scale bar: 1 µm). **(F)** Western blot analysis of protein lysates prepared from the lungs of Atg7^ SPC^ mice and littermate controls at P1. In Atg7^ SPC^ neonates, LC3B-I failed to convert to LC3B-II, and p62 was accumulated. All experiments were performed at least three times; representative data are shown. A quantitative analysis of SFTPB **(G)** and pro-SFTPC **(H)** (mice/group n ≥ 4). *P < 0.05; **P < 0.01; ***P < 0.001.

We then analyzed the lungs of* Atg7^SPC^* neonates (P1) and littermate mice with the genotype *Atg7*^flox/+^, *Atg7*^flox/+^:*(tetO)_7_-Cre*, and *Atg7^flox/+^:SPC-rtTA*. Because it has been reported that treatment of *SPC-rtTA* mice with doxycycline (DOX) may exert toxicity to alveolar epithelial cells 44, the phenotype of *Atg7^flox/+^:SPC-rtTA* mice was carefully compared with littermate controls. However, no apparent abnormality was observed in *Atg7^flox/+^:SPC-rtTA* mice under our experimental conditions, except for an enlarged alveolar cavity. H&E staining showed impaired thinning of the alveolar septa in *Atg7^SPC^* mice, whereas the lungs of control neonates showed normal septal thinning at birth (Figure 4A). Morphometric analysis revealed an increase in alveolar septal thickness in* Atg7^SPC^* mice (Figure 4B). Similar to our observations in *Atg7* KO mice, *Atg7^SPC^* mice had altered LBs (Figure 4C). Quantitative analysis revealed that the size of LBs in *Atg7^SPC^* mice was significantly reduced compared with control mice (Figure 4D). EM also revealed that the abnormal LBs in *Atg7^SPC^* mice had fewer lamellae than those in WT mice (Figure 4E). Furthermore, a substantial reduction in ATG7 protein expression was observed in* Atg7^SPC^* mice, sufficient to reduce autophagic activity, as evidenced by the increase in p62 and the decrease in the LC3B-II/LC3B-I ratio (Figure 4F). Western blot analysis of SFTPB and pro-SFTPC revealed reduced expression in* Atg7^SPC^* mice (Figure 4F-H). Collectively, these findings suggest that the phenotype observed in *Atg7* KO lungs is mainly due to the lack of ATG7 in AT2 cells.

### Postnatal Atg7^SPC^mice contain large and irregular ABCA3^+^ vacuoles

**Figure 5:**
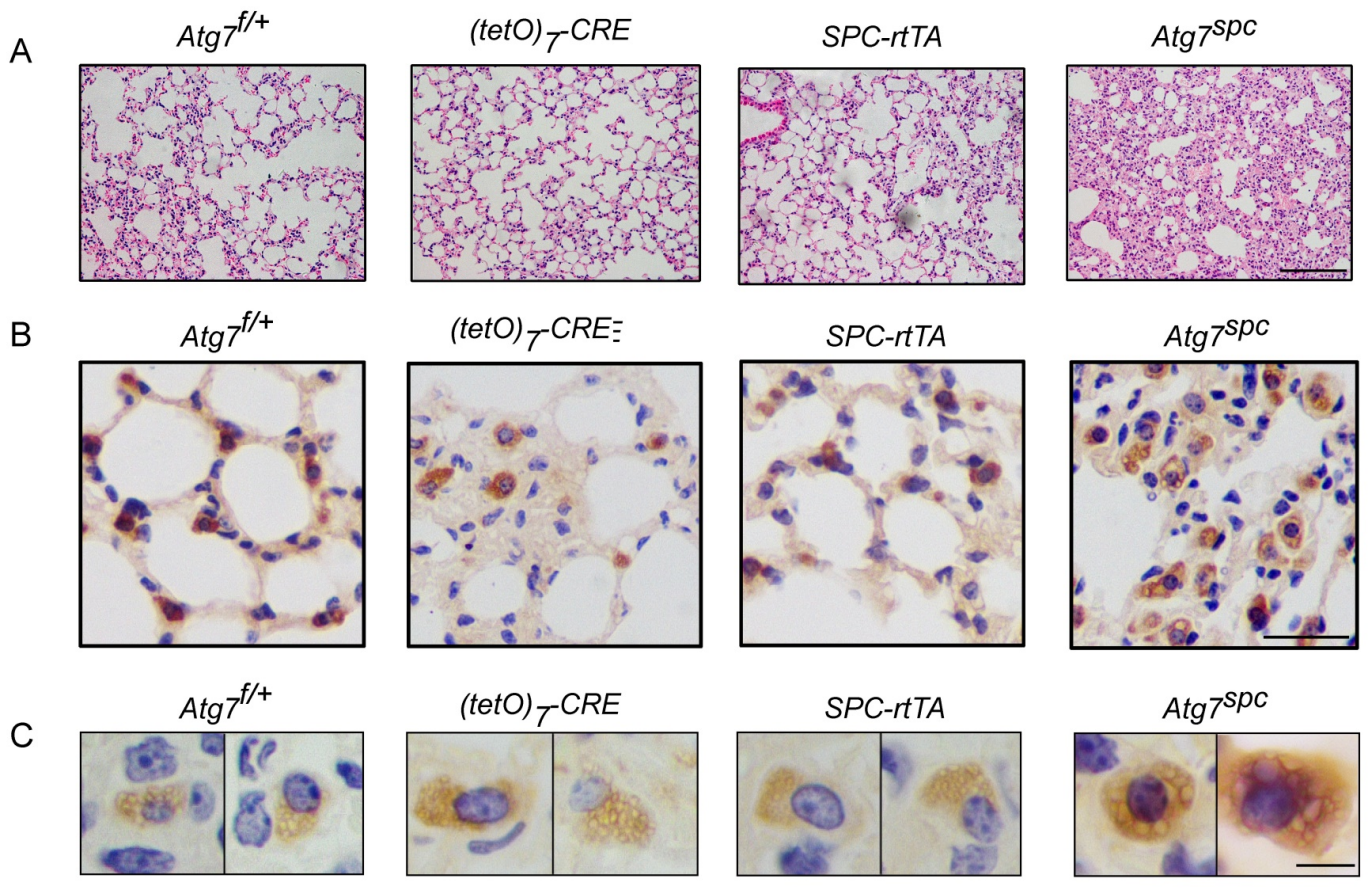
** Postnatal Atg7^SPC^ mice (P90) contain large and irregular ABCA3^+^ vacuoles. (A)** Histological analysis of lungs from control and Atg7^SPC^ mice (P90, scale bars: 100 µm). Immunostaining of Atg7^SPC^ lungs with anti-ABCA3 antibody shows swollen AT2 cells (scale bar: 20 µm) **(B)** and large and irregular ABCA3^+^ vacuoles, compared with littermate controls (scale bars: 5 µm) **(C)**.

To elucidate how ATG7 affects the lung postnatally, we performed microscopy and histological analyses of* Atg7^SPC^* mice at P90. Consistent with the observation in P1 mice, H&E staining revealed a thick lung septum (Figure 5A). Immunostaining of lung tissue with anti-ABCA3 antibody showed swelling of AT2 cells in *Atg7^SPC^* mice (Figure 5B). Additionally, this analysis showed LBs of uniform size in control mice, whereas the lung tissue from *Atg7^SPC^* mice contained large and irregular vacuoles (Figure 5C). Because these vacuoles were ABCA3-positive, we called them “ABCA3^+^ vacuoles” (LB-like vacuoles). Large LBs were also observed in patients with HPS, which is manifested with lower autophagic activity 28. Considering autophagosomes constantly fuse with LBs, these enlarged ABCA3^+^ vacuoles are probably a result of insufficient autophagic activity to maintain normal LBs. Collectively, these data indicate that *Atg7* regulates LB formation at the neonatal stage and maintains LB homeostasis at postnatal stages.

## Discussion

It is well-established that large amounts of glycogen accumulate in undifferentiated epithelial cells during lung development. The glycogen storage, which provides the substrates for synthesizing surfactant phospholipids and forming LBs, depletes as the pulmonary epithelium matures 6, 9, 10, 22. In the lungs of *Atg7* KO mice, the accumulation of glycogen indicated that the alveolar epithelial cells were defective in using cytoplasmic glycogen in the absence of autophagy. Examining the E18.5 lung, we found autolysosomes containing partially digested glycogen particles, which further confirmed the idea that glycogenolysis occurs through an autophagic process. Moreover, we found extensive fusion between autophagosomes and LBs in control mice (P1), especially in littermate control mice of *Atg^spc^* mice. These findings suggest that autophagy is essential for lung development, consistent with the critical role of glycogen autophagy in developing the liver and heart in newborn rats 45.

Currently, it is postulated that the MVB is the precursor of the LB. MVBs play a key role in delivering newly synthesized surfactant proteins and peptides that are internalized/recycled via the endocytic pathway to the LBs. Fluorescence microscopy revealed that autolysosomes could fuse with MVBs, particularly in the case of starvation or drug-induced autophagy 46. Moreover, the delivery of cytoplasmic components into the LBs was reversibly inhibited by 3-MA 8. Notably, LBs are similar in nature to the lysosome. Treatment of cells with the lysosomal protease inhibitor leupeptin resulted in the progressive transformation of LBs into electron-dense autophagic vacuoles and the eventual disappearance of LBs 8. Together with the observation of constant autophagosome-LB fusion, these results support the notion that LB is a specialized autolysosome.

Evidence suggests that activation of the PI3K*-*AKT*-*mTOR pathway in pulmonary epithelial cells may play a vital role in the pathogenesis of RDS in infants. The activation of AKT signaling in pulmonary epithelial cells results in transient respiratory difficulties in full-term and pre-term infants 47. These respiratory difficulties are associated with bronchiolar hyperplasia and maturation defects of pulmonary epithelial cells. Phosphatase and tension homolog deleted on chromosome 10 (PTEN), which inhibits PI3K, is highly expressed in the respiratory epithelium at the terminal sac stage, in which type 1 and type 2 alveolar cells differentiate. Specific deletion of *PTEN* in alveolar epithelium resulted in RDS, and approximately 90% of neonates died within 2 h after birth 48. Moreover, it has been reported that* PTEN* deletion had an adverse effect on LB formation 48. Although a previous study has demonstrated that the deletion of *Ulk1/2*, *Atg5*, *or Beclin 1* resulted in compromised lung development, LB formation was not reported to be altered 40, 43, 49. This discrepancy may be explained by functional differences among individual autophagic genes or different backgrounds. During our manuscript preparation, Mizushima's group showed that LBs were defective in AT2 cells in *Atg101-* and *Fip200-*deficient mice 50. Our results support their notion that autophagic vacuoles can fuse with LBs and autophagy is vital for LB growth. The reason why *Atg7^spc^* mice are viable is possibly due to a less critical role of ATG7 as a molecule in the conjunction system compared to upstream autophagic molecules such as FIP200 and ATG101. It cannot be excluded that the deletion efficiency might account for the difference in *Atg7* KO and* Atg7^spc^* mice in our study.

Taken together, our results provide fundamental insight into the role of autophagy in maintaining the proper function of LBs and AT2 cells. Furthermore, our study considerably improves our understanding of lung diseases, such as RDS and HPS, and will be of great significance in developing therapies for these diseases.

## Methods

### Mice

#### Global deletion mice

*atg7*^+/-^ mice were previously described 33. *atg7*^-/-^ mice were generated by mating *atg7*^+/-^ mice. Newborn pups (P1) were sacrificed after birth.

#### Generation of bronchioalveolar epithelium-specific Atg7-deficient mice SFTPC-rtTA/(tetO)7-Cre/Atg7^flox/flox^ mice

*SFTPC-rtTA* transgenic mice, expressing the reverse tetracycline transactivator (rtTA) protein under the control of the human *SFTPC* promoter, were described previously 51-53. The *SFTPC-rtTA* mice line was obtained from Jackson Laboratory with a C57BL/6J background. Transgenic *(tetO)_7_-Cre* mice 54, in which rtTA activated Cre expression, were obtained from Jackson Laboratory with a C57BL/6J background. *SFTPC-rtTA* transgenic mice were mated to *tetO-Cre* mice to generate *SFTPC-rtTA;tetO-Cre* mice. The double transgenic mice were further mated with *Atg7*^flox/flox^ mice (C57BL/6J background) 24 to generate triple-transgenic mice. To induce expression of the Cre transgene, mice were administrated doxycycline in utero. Specifically, dams bearing pups were fed doxycycline in their drinking water (1 mg/mL) at least seven days before delivery.

### Histological analysis

Neonate mice were immediately sacrificed after birth by neck removal. Lung tissue was perfused with PBS and fixed in 4% buffered formalin overnight, followed by dehydration, embedding, and sectioning following standard protocols. Sections (5 μm) were mounted on slides for H&E staining. Aerated lung areas and alveolar septal thickness were measured in H&E stained sections using Nikon NIS-Elements BR Analysis software. Septal thickness was measured at all points in each visual field, and five visual fields for each animal were used for the measurement. According to the manufacturer's instructions, sections were also stained with Periodic Acid Schiff stain (PAS, Sigma-Aldrich, St. Louis, MO, #395) to visualize intracellular glycogen. For immunohistochemical staining, tissue sections were deparaffinized and then rehydrated in graded ethanol from 100% to 75%. For antigen retrieval, sections were incubated with 0.1% proteinase K (#19131, Qiagen) for 15 min at room temperature. After washing with PBS, sections were subjected to UltraSensitiveTM SP (Mouse/Rabbit) IHC Kit according to the manufacturer's instructions (MXB KIT-9720, MXB Biotechnologies, China). Brown signals were visualized using DAB (MXB 0031, MXB Biotechnologies, China). Slides without primary antibodies were used as negative controls.

### TEM

Mouse lungs were freshly dissected, finely minced into millet size, and fixed in 2.5% glutaraldehyde in PBS at 4 °C. Samples were examined by TEM (JEM-1200EX, JEOL, Japan). Autophagic vacuoles, LBs, and glycogen were identified by visual inspection of EM micrographs using established criteria 13, 29, 55, 56. To quantify the size of LB in EM, ImageJ software was used. In 10,000 magnification fields, the size of at least 80 LBs/mouse was measured and averaged. To quantify the number of LBs, at least 25 AT2 cells/mouse were quantified.

### Isolation of primary AT2 cells from neonate and adult mice

Primary AT2 cells from adult mice were isolated as previously described 57, and primary AT2 cells from neonate mice were isolated similarly with moderate modifications. Neonate mice (P1) were perfused with PBS through the right ventricle of the heart until visually free of blood. The trachea was exposed by clearing surrounding tissue. Dispase was injected into the lung through the trachea by an insulin syringe, and the lung was allowed to collapse for 5 min. Lungs were removed from the animal and put in a tube containing 1mL of dispase. Lungs were minced and incubated on a shaker at room temperature for 45 min. After centrifugation, the isolating procedure followed Sun *et al*. 57.

### Isolation and adenoviral infection of primary human AT2 cells

Primary AT2 cells from human lungs were isolated as previously described 58. For immunofluorescent staining, isolated cells were seeded into Nunc^TM^ Lab-TEK^TM^ Chambered Coverglass (Thermofisher Scientific). 70 nM of LysoTracker (L7528, Thermofisher Scientific) was added two hours before cell collection. Adenoviruses with *ATG7* shRNA plasmid constructed into an adenoviral vector (pAdEasy-U6-CMV-EGFP) were purchased from Hanbio Technology Ltd. (Shanghai, China). The shRNA sequence used to target human *ATG7* (sh-*ATG7*) was 5'- GCCTGCTGAGGAGCTCTCCAT -3', and the control sequence was 5'- TTCTCCGAACGTGTCACGTAA -3'. The titter of adenoviruses was 3.16×10^10^ PFU/mL. The multiplicity of infection was 100:1. Cells were collected for immuno-staining 48 hours after infection.

### Glycogen assay

Glycogen assay kits were purchased from Sigma-Aldrich (#MAK016), and the assays were performed according to the manufacturer's instructions. The relative glycogen level was calculated by comparing it with the absorbance from the standard.

### Western Blot analysis

Frozen lung tissues were sonicated in RIPA lysis buffer (50 mM Tris-HCl, pH 8.0, 150 mM NaCl, 1% NP-40, 0.5% deoxycholate, 0.1% SDS, 1 mM EDTA) containing protease inhibitor cocktail (Roche Applied Bioscience). Protein content was determined by Bradford assay (Amresco) staining using bovine serum albumin as a standard. Equal amounts of protein were loaded on 10%, 12%, or 15% Tris-glycine gels for electrophoresis. Proteins were wet-transferred to PVDF membranes (Millipore) and then probed with the indicated antibodies. The primary antibodies and dilutions used were as follows: anti-surfactant protein B rabbit polyclonal antibody (1:3,000, #07-614, Millipore), anti-surfactant protein C rabbit polyclonal antibody (1:1000, #AB3786, Millipore), anti-surfactant protein A rabbit polyclonal antibody (1:1000, #AB3420, Millipore), anti-p62 rabbit polyclonal antibody (1:1000, #P0067, Sigma), LC3B rabbit polyclonal antibody (1:1000, #12741, Cell Signaling Technology), anti-ATG7 rabbit polyclonal antibody (1:1000, #A2856, Sigma) and anti-actin mouse polyclonal antibody (1:1000, #TA-09, Zsgb-bio, China). Immunoreactivity was detected using horseradish peroxidase-conjugated secondary antibodies. Chemiluminescence substrates were used (Tiangen, Beijing, China). The images were captured using a MicroChemi 4.2 system (DNR Bio Imaging Systems, Jerusalem, Israel).

### Immunofluorescence and confocal microscopy

Lung tissues were fixed with 4% paraformaldehyde for 30 min and cryopreserved by sequential sucrose incubation. Tissues were embedded in OCT medium, and 4 µm sections were cut and mounted on slides. Slides were air-dried and stored at -80 °C. For immunostaining, sections were fixed with 4% paraformaldehyde for 15 mins and permeabilized with 0.5% Triton X-100 for 15 mins. Following permeabilization, sections were blocked with 10% normal horse serum in PBS supplemented with 0.1% Triton X-100 for 1 h at room temperature and then incubated overnight at 4 °C with rabbit polyclonal anti-SFTPB (1:2000, #07-614, Millipore), mouse polyclonal anti-ABCA3 (1:200, #AB24751, Abcam), anti-pro-SFTPC (1:200, #3786, Millipore) or LC3B (1:200, #18725-1-AP, Proteintech). Slides were washed with PBS, incubated with Alexa Fluor-conjugated secondary antibodies (Alexa Fluor-488 or Alexa Fluor-594, Life Technologies), stained with DAPI (Life Technologies). For co-labeling experiments, AT2 cells were isolated from neonate mice (P1), centrifuged using cytospin (Thermo Scientific), and stained with antibodies as needed. Slides were imaged on a Nikon A1R confocal microscope. We conducted fluorescence co-localization analyses using NIH Image J software. Pearson correlation coefficient (PCC) was calculated by Image J scatter J plug-in. The line profile plots were performed as indicated. PCC was calculated from the region of interest.

### Rapamycin or 3-MA treatment

Rapamycin (#53123-88-9, BioChemPartner, China) was initially dissolved in 100% DMSO, stored at -20 °C, and further diluted in PBS immediately before use. WT adult mice (eight-week-old) were randomly divided into two groups. Mice were intraperitoneally injected with rapamycin (40 μg/kg) or vehicle. Eight hours later, mice were harvested. Eight-week-old mice were intraperitoneally injected with 20 mg/kg/day 3-MA (#M9281, sigma) or vehicle for five consecutive days. Primary human AT2 cells were treated with rapamycin or vehicle control for eight hours. Primary human AT2 cells were treated with 3-MA (15 mM) or vehicle control for 24 hours. 70 nM of LysoTracker was added two hours before cell collection.

### Statistics

Data analyses and the construction of statistical charts were performed using GraphPad Prism 5 software (GraphPad Software, CA, USA). The results were presented as the mean value (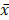 ± SEM). Statistical significance was determined using an independent samples *t*-test. A value of *P* < 0.05 was considered statistically significant. For western blots, at least three experiments were performed, and gels were quantified using ImageJ software.

### Study approval

All animal experiments were approved by the animal experiment review board of the China Medical University (Ethics approval number 16099M). The study of isolation of human primary AT2 cells was approved by the Research Ethical Board of China Medical University [2017]074 for the use of human lungs from distal portions of normal lung tissue from patients undergoing lung resection.

## Supplementary Material

Supplementary methods, figures and table.

## Abbreviations

LB: lamellar body
AT2: alveolar type 2 cell
WPBs: Weibel-Palade bodies
LC3B (MAP1LC3B): microtubule-associated protein 1 light chain 3B
*Atg7*: autophagy-related 7
MVB: multivesicular body
TEM: transmission electronic microscopy
SFTPB: surfactant protein B
SFTPC: surfactant protein C
LROs: lysosome-related organelles
HPS: Hermansky-Pudlak Syndrome
3-MA: 3-methyladenine
RDS: respiratory distress syndrome
ABCA3: ATP-binding cassette sub-family A [ABC1]: member 3
mTOR: mechanistic target of rapamycin kinase
ULK1: UNC-51-like kinase 1
*Atg7* KO: *Atg7* deficient mice
mTOR: mechanistic target of rapamycin kinase
Avs: autophagic vacuoles
PTEN: phosphatase and tension homology
