# Supplementary material for: The Role of Autophagy in Lamellar Body Formation and Surfactant Production in Type 2 Alveolar Epithelial Cells
Source: Int J Biol Sci. 2022 Jan 1;18(3):1107–19. doi: 10.7150/ijbs.64285 (PMC8771840; doi:10.7150/ijbs.64285)
Supplement: Supplementary file 1 — Supplementary methods, figures and table. [file ijbsv18p1107s1.pdf]

Supplemental methods

Lipid quantification

WT and *Atg7* KO lungs were homogenized with a steel ball and 1 mL of a solution containing methanol, methyl tert-butyl ether, and an internal standard mixture. Then, 200 µl of water was added, and the mixture was whirled for 1 min, followed by centrifugation at 12,000 rpm at 4°C for 10 min. Three hundred microliters of the supernatant were collected and concentrated. The pellet was resuspended in 200 µL of reconstitution solution and subjected to LC-ESI-MS/MS (UPLC, ExionLC AD; MS, QTRAP® 6500+System). The analytical conditions were: UPLC: column, Thermo Accucore™C30 (2.6 µm, 2.1 × 100 mm i.d.); solvent system, A: acetonitrile/water (60/40, V/V, 0.1% formic acid, 10 mmol/L ammonium formate), B: acetonitrile/isopropanol (10/90 VV/V, 0.1% formic acid, 10 mmol/L ammonium formate); gradient program, A/B (80:20, V/V at 0 min, 70:30 V/V at 2.0 min, 40:60 V/V at 4 min, 15:85 V/V at 9 min, 10:90 V/V at 14 min, 5:95 V/V at 15.5 min, 5:95 V/V at 17.3 min, 80:20 V/V at 17.3 min, 80:20 V/V at 20 min); flow rate, 0.35 mL/min; temperature, 45°C; injection volume: 2 µL. The effluent was alternatively connected to an ESI-triple quadrupole-linear ion trap (QTRAP)-MS. Significantly regulated metabolites between groups were determined by VIP ≥ 1, absolute Log_2FC (fold change) ≥ 1, and $P < 0.05$. VIP values were extracted from the OPLS-DA (MetaboAnalystR) result, which contains score plots and permutation plots, generated using the R package MetaboAnalystR. The data were log-transformed (log2) and

mean-centered before OPLS-DA. To avoid overfitting, a permutation test (200 permutations) was performed.

Supplemental Figures

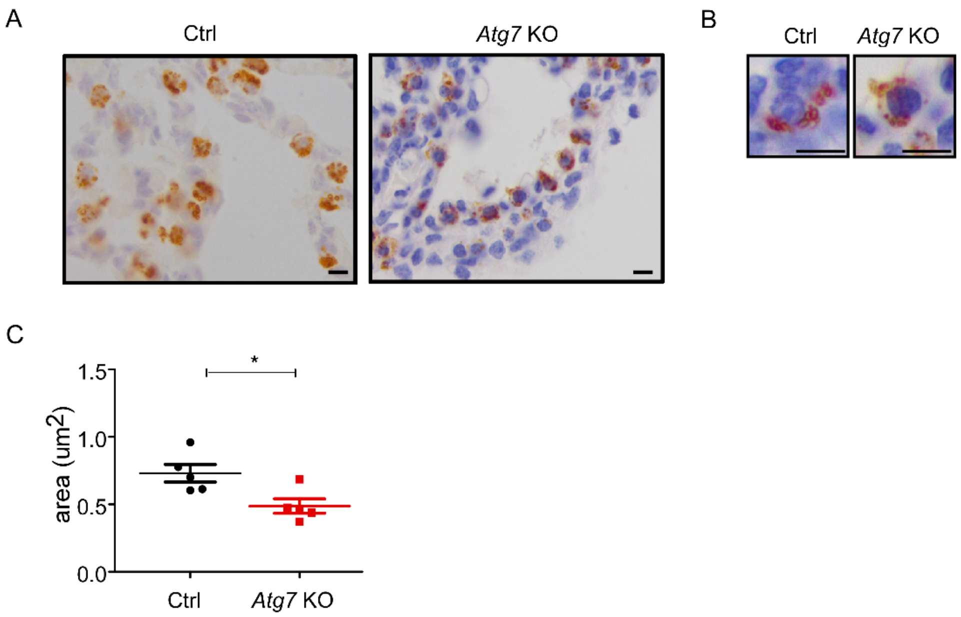

Supplemental Figure 1. (**A**) Immunohistochemical staining with anti-ABCA3 antibody was used to visualize the limiting membrane of the LB in control and *Atg7* KO mice (scale bar: 5 µm). (**B**) Images showing the LB size in the control mice was larger than that in the *Atg7* KO mice (scale bar: 5 µm). (**C**) The average area of LBs in control and *Atg7* KO mice (80 LBs in at least 5 high magnification fields were measured. WT, n = 5; KO, n = 5). *$P < 0.05$.

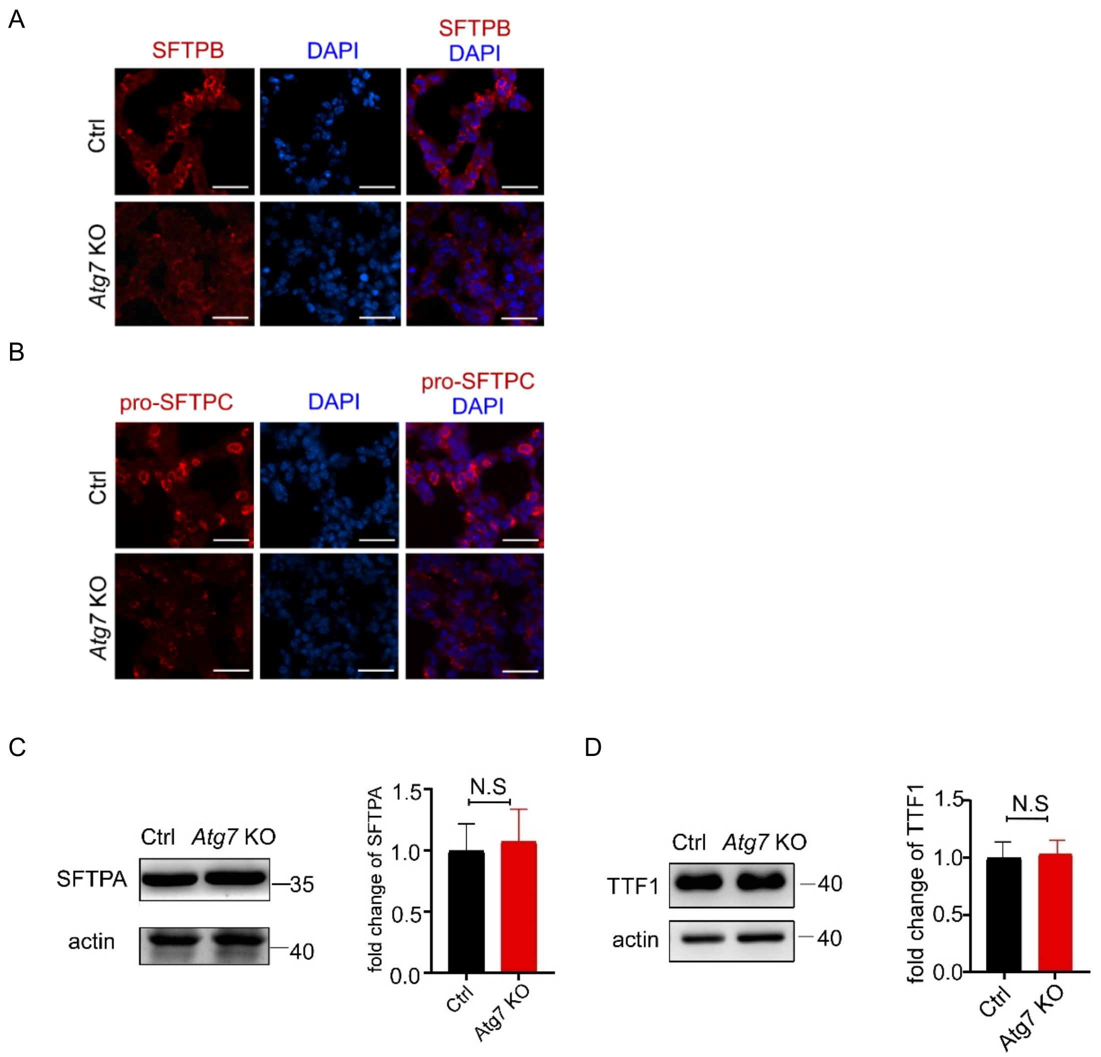

Supplemental Figure 2. (A) Immunofluorescent staining confirmed the reduction in SFTPB expression in *Atg7* KO mouse lungs (scale bar: 20 μm). **(B)** Immunofluorescent staining showed a decrease of pro-SFTPC expression in *Atg7* KO mouse lungs (scale bar: 20 μm). **(C)** Western blot analysis showed that SFTPA was not altered in the lung homogenates of *Atg7* KO mice, compared with the WT mice (n = 5 mice/group). **(D)** Western blot analysis showed that TTF-1 was not altered in the lung homogenates of *Atg7* KO mice, compared with the WT mice (n = 5 mice/group).

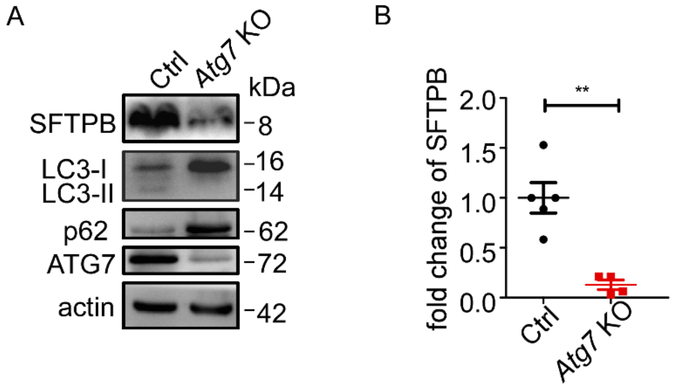

Supplemental Figure 3. (**A**) The levels of SFTPB in extracts of AT2 cells isolated from control and *Atg7* KO mice were determined by western blotting. Experiments were repeated at least three times; representative images are shown. (**B**) Densitometry analysis of SFTPB from control and *Atg7* KO mice (WT, n = 5; KO, n = 4). $^{**}P <$ 0.01.

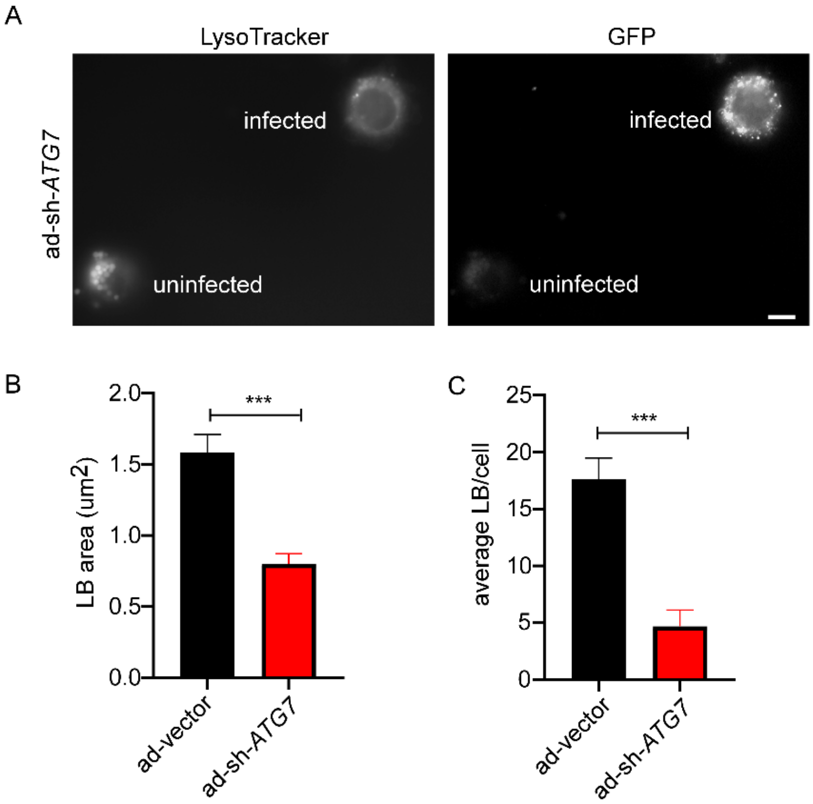

Supplemental Figure 4. (**A**) Knockdown of *ATG7* by adenoviral infection inhibited LB formation in human AT2 cells. In cells treated with ad-sh-*ATG7,* successfully infected cells have GFP-fluorescence but lack lysotracker staining; uninfected cells have lysotracker staining but lack GFP-fluorescence (scale bar: 5 μm). (**B**) The average area of LBs in cells infected with ad-sh-*ATG7* was plotted against cells infected with adenoviral-vector (80 LBs in at least 5 random fields were measured, ****P* < 0.001). The size of LBs was measured using ImageJ software and plotted using GraphPad Prism 5. (**C**) The average number of LBs in cells infected with ad-sh-*ATG7* or adenoviral-vector (at least 25 random AT2 cells were chosen, ****P* < 0.001).

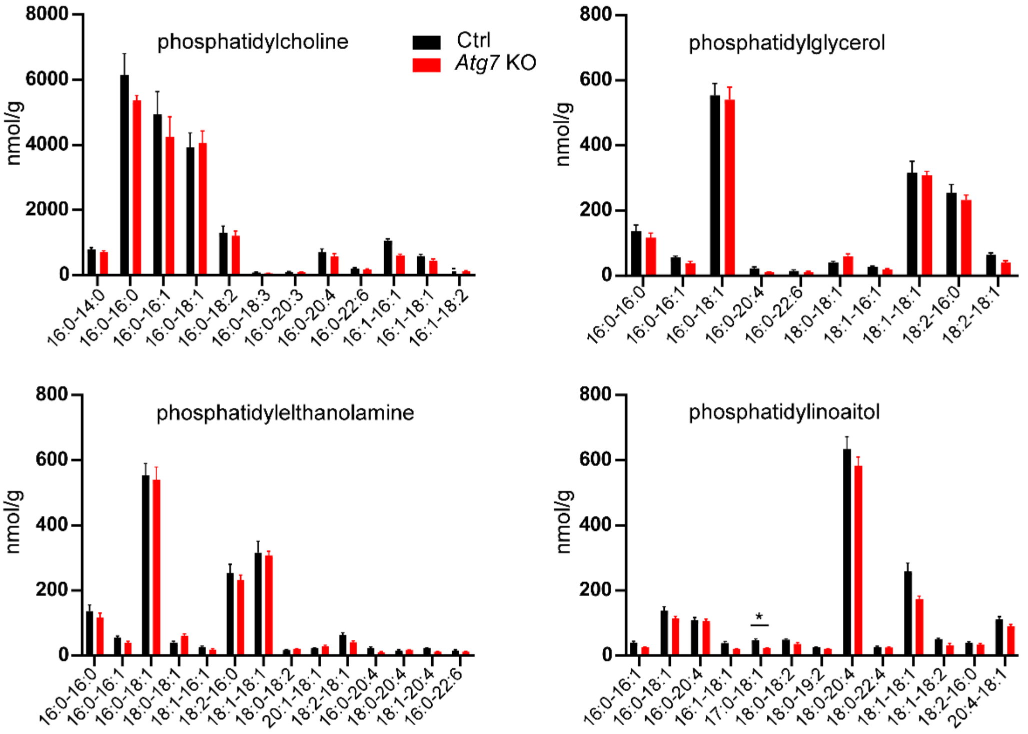

Supplemental Figure 5. LC-MS/MS analysis of the number of indicated phospholipids. The y-axis indicates nmol/g neonate P1 lung tissue (WT, n=5; *Atg7* KO, n=3).

Supplemental Table 1

Compounds	*P* value	Fold of change	Type of change in KO
lysophosphatidylcholine (0:0/18:0)	0.0015	0.3528	down
lysophosphatidylcholine (24:1)	0.0016	0.4637	down
phosphatidylcholine (16:1/18:3)	0.0037	0.3518	down
phosphatidylcholine (20:4/20:4)	0.0131	0.4616	down
phosphatidylglycerol (16:1/20:4)	0.0040	0.4735	down
phosphatidylglycerol (18:1/20:4)	0.0048	0.4885	down
phosphatidylglycerol (16:1/16:1)	0.0116	0.4811	down
lysophosphatidylglycerol (22:4)	0.0338	0.4893	down
phosphatidylglycerol (16:0/20:4)	0.0490	0.4753	down
lysobisphosphatidic acids (22:6)	0.0108	0.4547	down
phosphatidylinositol (17:0/18:1)	0.0082	0.4951	down
phosphatidylinositol (18:1/22:6)	0.0127	0.4157	down
phosphatidylserine (17:0/16:1)	0.0149	0.421	down
phosphatidylethanolamine (18:3/16:1)	0.0352	0.4498	down

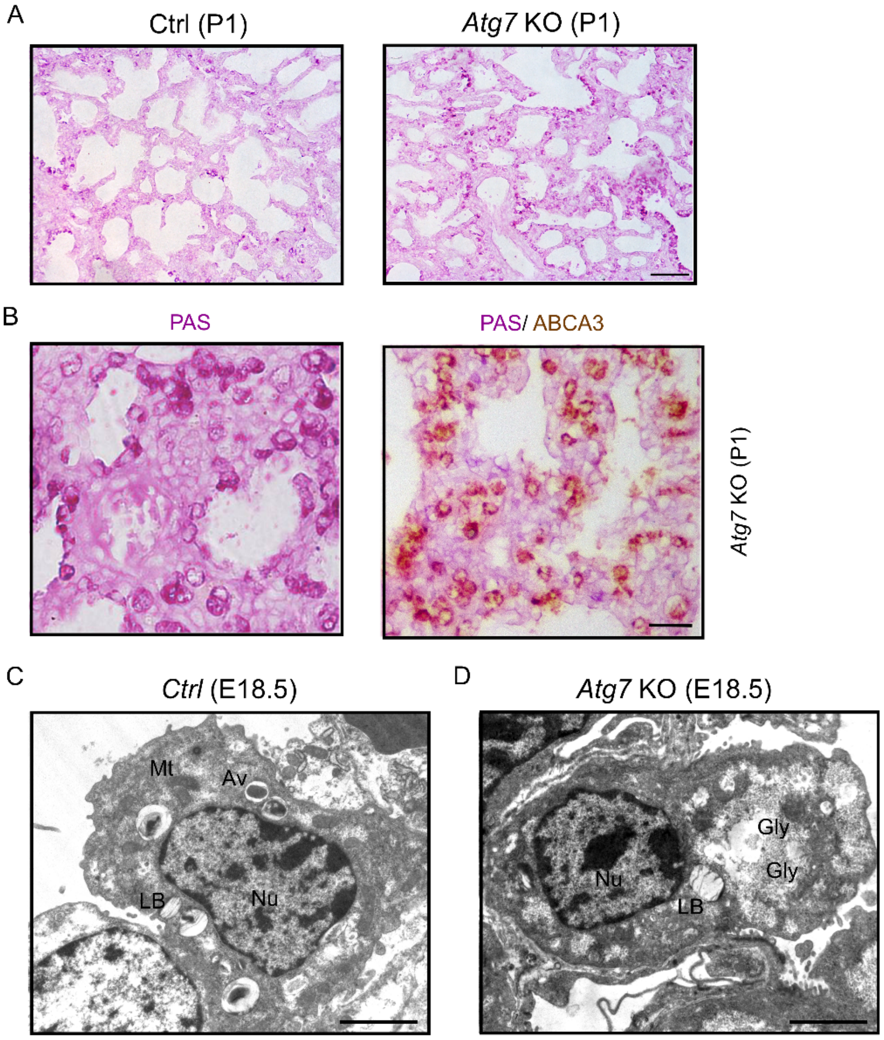

Supplemental Figure 6. (A) PAS staining of lung sections from *ATG7* KO mice at P1 shows an abnormal presence of glycogen-filled cells (scale bar: 50 µm). **(B)** PAS staining was mainly located in AT2 cells. Lung transverse sections from *Atg7* KO mice (P1) were immunostained with anti-ABCA3 antibody followed by PAS staining (scale bar: 20 µm). **(C, D)** EM images of an E18.5 WT and *Atg7* KO mouse lung. WT mice showed autophagic vacuoles, and *Atg7* KO mice showed sequestered glycogen but without a membrane (Av denotes autophagic vacuoles, Mt denotes mitochondria, Gly denotes glycogen, Nu denotes nucleus; scale bar: 2 µm).
